# Impact of Oncoming Headlight Glare With Cataracts: A Pilot Study

**DOI:** 10.3389/fpsyg.2018.00164

**Published:** 2018-03-06

**Authors:** Alex D. Hwang, Merve Tuccar-Burak, Robert Goldstein, Eli Peli

**Affiliations:** Schepens Eye Research Institute, Massachusetts Eye and Ear, Department of Ophthalmology, Harvard Medical School, Boston, MA, United States

**Keywords:** cataracts, headlight glare, driving simulator, headlight glare simulator, visual performance, low vision

## Abstract

**Purpose:** Oncoming headlight glare (HLG) reduces the visibility of objects on the road and may affect the safety of nighttime driving. With cataracts, the impact of oncoming HLG is expected to be more severe. We used our custom HLG simulator in a driving simulator to measure the impact of HLG on pedestrian detection by normal vision subjects with simulated mild cataracts and by patients with real cataracts.

**Methods:** Five normal vision subjects drove nighttime scenarios under two HLG conditions (with and without HLG: HLGY and HLGN, respectively), and three vision conditions (with plano lens, simulated mild cataract, and optically blurred clip-on). Mild cataract was simulated by applying a 0.8 Bangerter diffusion foil to clip-on plano lenses. The visual acuity with the optically blurred lenses was individually chosen to match the visual acuity with the simulated cataract clip-ons under HLGN. Each nighttime driving scenario contains 24 pedestrian encounters, encompassing four pedestrian types; walking along the left side of the road, walking along the right side of the road, crossing the road from left to right, and crossing the road from right to left. Pedestrian detection performances of five patients with mild real cataracts were measured using the same setup. The cataract patients were tested only in HLGY and HLGN conditions. Participants’ visual acuity and contrast sensitivity were also measured in the simulator with and without stationary HLG.

**Results:** For normal vision subjects, both the presence of oncoming HLG and wearing the simulated cataract clip-on reduced pedestrian detection performance. The subjects performed worst in events where the pedestrian crossed from the left, followed by events where the pedestrian crossed from the right. Significant interactions between HLG condition and other factors were also found: (1) the impact of oncoming HLG with the simulated cataract clip-on was larger than with the plano lens clip-on, (2) the impact of oncoming HLG was larger with the optically blurred clip-on than with the plano lens clip-on, but smaller than with the simulated cataract clip-on, and (3) the impact was larger for the pedestrians that crossed from the left than those that crossed from the right, and for the pedestrians walking along the left side of the road than walking along the right side of the road, suggesting that the pedestrian proximity to the glare source contributed to the performance reduction. Under HLGN, almost no pedestrians were missed with the plano lens or the simulated cataract clip-on (0 and 0.5%, respectively), but under HLGY, the rate of pedestrian misses increased to 0.5 and 6%, respectively. With the optically blurred clip-on, the percent of missed pedestrians under HLGN and HLGY did not change much (5% and 6%, respectively). Untimely response rate increased under HLGY with the plano lens and simulated cataract clip-ons, but the increase with the simulated cataract clip-on was significantly larger than with the plano lens clip-on. The contrast sensitivity with the simulated cataract clip-on was significantly degraded under HLGY. The visual acuity with the plano lens clip-on was significantly improved under HLGY, possibly due to pupil myosis. The impact of HLG measured for real cataract patients was similar to the impact on performance of normal vision subjects with simulated cataract clip-ons.

**Conclusion:** Even with mild (simulated or real) cataracts, a substantial negative effect of oncoming HLG was measurable in the detection of crossing and walking-along pedestrians. The lowered pedestrian detection rates and longer response times with HLGY demonstrate a possible risk that oncoming HLG poses to patients driving with cataracts.

## Introduction

More than 25% of people over the age of 55 in the United States develop cataracts (Acosta et al., 2006), and this figure grows to more than 50% for people over the age of 65 (Klein et al., 1992). Cataracts reduce visual acuity and contrast sensitivity, causing patients with advanced cataracts to fail the visual acuity requirements for a driver’s license. However, mild, early stage cataracts may have little impact on visual acuity (Adamsons et al., 1992; Elliott and Situ, 1998), typically remaining at 20/40 or better (Owsley et al., 2008), meeting the legal requirements for unrestricted driving at day and night. The safety of driving with cataracts remains in question, as indicated by epidemiological studies, which have shown that older drivers with cataracts have higher crash involvement than similarly aged drivers without cataracts (Owsley and McGwin, 1999; Owsley et al., 2002). Those epidemiological studies, however, did not separate daytime from nighttime driving records, and for night accidents, the study did not separate crashes involving highly visible self-illuminated objects (vehicles) from non-light-emitting road users (pedestrians and animals).

Oncoming headlight glare (HLG) may affect driving in two ways. (1) The bright light from the oncoming headlights scatters within the eyes, directly reducing retinal image contrast (veiling glare) and thus reducing overall visibility (*disability glare*). This visibility reduction may impair performance on visual tasks related to driving safety (e.g., detecting pedestrians, animals and other on-road objects, or following lane margins). (2) The visual distraction and annoyance of light sensitivity (photophobia) caused by the bright light results in discomfort (*discomfort glare*), which may affect driving by causing changes in driver’s eye or head movements, and consequently affect steering (Readinger et al., 2002; Chattington et al., 2007) and visibility of other road users due to the change in eccentricity (Bronstad et al., 2013).

Studies of police crash reports found that oncoming HLG is rarely reported as a major factor in nighttime driving accidents (Owsley and McGwin, 1999; Owsley et al., 2002) when compared to low visibility, alcohol, and sleep. Yet, the HLG involvement in nighttime accidents was estimated at between 0.5 and 4.0% (Hermion, 1969), and considered to be at least a contributing factor for about 0.3% of fatal accidents at night (National Highway Traffic Safety Administration [NHTSA], 2007). The public concern of HLG as a driving risk factor is widespread, generating more than 5,000 online comments in 3 months, after the National Highway Traffic Safety Administration (NHTSA) opened a forum (NHTSA, 2001) to identify the headlight glare related concerns of United States drivers. A focus group study showed that older drivers are likely to perceive HLG as their primary nighttime driving concern (Mace et al., 2001). Since glare is a visual effect caused by light scatter (Miller and Benedek, 1973; Van den Berg, 1991) and cataracts increase light scatter within the eye (Sjostrand et al., 1987; de Waard et al., 1992; Regan et al., 1993), it is expected that the driving risks due to oncoming HLG will be higher for cataract patients, as cataracts may affect detection of and response to road hazards.

Wood and Carberry (2006) conducted a daytime closed-circuit driving performance study, and found that bilateral cataract patients performed significantly worse than the normal vision control group on driving related tasks (e.g., sign and hazard detection and recognition, hazard avoidance, and gap perception). Their performance improved to the level of normal vision subjects after both cataracts were removed. Similar results were found in video based hazard perception and detection tests with two different levels of simulated cataract goggles (Marrington et al., 2008).

Nighttime driving conditions and the associated impact of oncoming HLG make driving at night more challenging (and dangerous) than daytime driving. A nighttime driving study (Wood et al., 2010) found that driving performance in a closed circuit was better predicted by contrast sensitivity than visual acuity. In that study, the driving performance of subjects with simulated cataract goggles and visual acuity-matching optically blurred goggles was compared. The simulated cataract group hit about twice as many hazards (traffic cones) as the visual acuity matched optically blurred group, who, in turn, hit about twice the number of hazards as the normal vision control group. In that study, two sets of headlamps were placed along the driving course to simulate HLG, but road hazards/pedestrians were not specifically placed relative to the glare sources, so the results did not directly address the effect of HLG.

In a second nighttime driving study (Wood et al., 2012), the pedestrians were positioned near the glare sources (2.5 m away from the stationary glare source) to measure the impact of HLG. A decline in the average detection rate of the static pedestrians with HLG was observed with the simulated vision impairments; simulated cataract (30%) and optically blurred (52%) compared to the normal vision condition (57%), which was similar to their previous daylight study (Wood et al., 2010). The overall detection rate for pedestrians in the presence of HLG was about half the detection rate without HLG, and the performance decreased more with simulated cataract. However, the pedestrians in that study were stationary with three different clothing and motion conditions: black shirts, retroreflective vest, and retroreflective vest with biomotion (walked in place). Therefore, that study tested the conspicuity of road workers with a fixed work light source, and did not directly address typical pedestrian activities (e.g., crossing and walking along the road) which represent the majority of nighttime accidents involving pedestrians (Sullivan and Flannagan, 2007; Chang, 2008; National Highway Traffic Safety Administration [NHTSA], 2016). Note that in dynamic pedestrian activities, the angular proximity of the pedestrian to the oncoming car varies while the oncoming car passes by, but in the static pedestrian activity condition, the pedestrian proximity to the glare source does not change much until the very last moment when the driver’s car passes by the glare source and the pedestrian.

A series of studies done by Ginsburg and Kelly (1995, Ginsburg, 2004) measured the detection and identification distance of road signs and hazards under various conditions (i.e., fog and HLG) using a nighttime driving simulator (NDS). The NDS is composed of a rear projection of a night driving video, shot from a car, without active driving. The system was also used for measuring the effect of refractive surgery on nighttime driving performance (Ginsburg and Subramaniam, 2007; Schallhorn et al., 2009). However, since the NDS only simulates the HLG from the following car, where the headlights are reflected in the rear view and side mirrors, it did not address the risk of HLG from an oncoming car. Note that the brightness of HLG from a following car can be easily reduced by adjusting mirror angles, or by auto dimming side/rear view mirrors. Tinted windows help to reduce the glare from HLG reflected on the mirrors, but the tint cannot be applied to the front windshield, so no practical solution currently exists for reducing oncoming HLG. Although those studies included a few pedestrian detection events, a major portion of the NDS test relied on sign readability, which becomes less problematic during real night driving because retroreflective signs become very high contrast with the driver’s car headlights.

Although prior studies provided important indications of the impact of cataracts and HLG on overall nighttime driving performance, they did not measure the direct impact of oncoming HLG on driving with cataracts, especially in critical conditions such as when pedestrians are crossing the road or walking alongside the road.

In our study, we utilized a novel custom HLG simulator (Hwang and Peli, 2013) for our driving simulator, which dynamically overlays bright programmable LED lights over the virtual oncoming car’s headlight positions (using a beam splitter placed between the monitor of the driving simulator and driver), while matching the temporal variation of the real-world car headlight luminance. We measured the impact of oncoming HLG on the detection of pedestrians in realistic situations on normal vision subjects with simulated cataracts and on real cataract patients, in preparation for a study on the impact of cataract surgeries for patients with bilateral cataracts on nighttime driving performance.

## Materials and Methods

### Ethics Statement

This study protocol (14-080H) was approved by the Massachusetts Eye and Ear Infirmary (MEEI)’s Institutional Review Board (IRB), and carried out in accordance with the recommendations of the ethical principles for medical research involving human subjects with written informed consent from all subjects. All subjects gave written informed consent in accordance with the Declaration of Helsinki.

### Participants and Viewing Conditions

The subjects’ binocular vision and driving performance were measured in the driving simulator in a dark room with the dynamic HLG simulator turned on (HLGY) and off (HLGN). The measurements for the five normal vision subjects were repeated under three (simulated) vision conditions: habitual correction with the plano lens clip-on, simulated cataract clip-on, and optically blurred clip-on. For the simulated cataract clip-on, a 0.8 Bangerter foil (Ryser Ophthalmology, Gallen, Germany) was applied to the front surfaces of a plano clip-on to simulate the effects of mild cataracts (Odell et al., 2008; Perez et al., 2010). The foil was selected to reduce the distance visual acuity (measured in the clinic with dark letters on bright background) to about 20/35 (0.24 LogMAR). The visual acuity requirements in Massachusetts, United States for driving in both daylight and nighttime are 20/40, and 20/70 for daylight only. The optically blurred clip-on’s (positive) dioptric power was individually fitted to approximately match each subject’s visual acuity through the simulated cataract clip-on at the driving simulator (measured without HLG in a dark room with bright letters on dark background), as described below.

Five patients with mild real cataracts who were considering cataract surgery in the near future were also recruited. The driving performance of the real cataract patients was measured using the same setup, but the patients were only tested for the two HLG conditions (HLGY/HLGN). **Table 1** reports participants’ age and visual functions as measured following standard clinical protocols.

**Table 1:** Vision information of the normal vision (NV) subjects and real cataract (RC) patients, measured by using standard clinical protocols.

Subject	Age	Habitual Distance VA		Letter CS		Habitual Spectacle SERx^∗^	
	[Years]	[Snellen (decimal)]		[Log CS]		[Diopter]	
		Right eye	Left eye	Right eye	Left eye	Right eye	Left eye
NV1	39	20/24 (0.83)	20/23 (0.87)	1.70	1.70	-7.25	-6.75
NV2	28	20/17 (1.18)	20/16 (1.25)	1.80	1.80	Plano	Plano
NV3	30	20/24 (0.83)	20/24 (0.83)	1.75	1.75	-5.00	-7.75
NV4	29	20/18 (1.11)	20/20 (1.00)	1.95	1.80	-1.75	-2.00
NV5	30	20/17 (1.18)	20/18 (1.11)	1.90	1.85	-7.75	-5.00

RC1	75	20/69 (0.29)	20/17 (1.18)	1.50	1.75	Plano	Plano
RC2	67	20/22 (0.91)	20/26 (0.77)	1.65	1.70	+1.00	+1.00
RC3	72	20/76 (0.26)	20/42 (0.48)	1.65	1.50	-0.75	-0.50
RC4	64	20/38 (0.53)	20/36 (0.56)	1.50	1.65	-1.00	-1.50
RC5	65	20/36 (0.56)	20/30 (0.67)	1.65	1.50	+4.00	+4.50

^∗^*SERx, spherical equivalent prescription*.

### Measuring Visual Acuity and Contrast Sensitivity in a Driving Simulator

Before nighttime driving performance measurements, the visual acuity and contrast sensitivity of each participant was measured with our mobile visual acuity and contrast sensitivity measuring smart phone app (Hwang and Peli, 2016), which enables us to measure visual acuity and contrast sensitivity in positive contrast polarity (a bright letter on dark background). The luminance for the dark background of the contrast sensitivity and visual acuity measuring app was 0.39 cd/m^2^. The luminance for letter stimulus was 329 cd/m^2^ for visual acuity measures and varied between 0.39 cd/m^2^ and 329 cd/m^2^ for contrast sensitivity measures. This app ran on a mobile phone (LG Optimus G Pro) mounted on the dashboard of the driving simulator. The viewing distance for visual acuity and contrast sensitivity measures varied slightly for each participant (0.64–0.74 m) because each participant adjusted the seat distance for comfortable driving. The viewing distance to the screen was measured, and then the measuring app was reconfigured to reflect this individual setting.

A stationary nighttime scenario was developed for the visual acuity and contrast sensitivity measures. The scenario simulated a car parked on the opposite side of a two-lane road (4 m width each lane), 60 m away from the participant’s car. The target letters of the measuring app were aligned with the center of the participant’s car lane. Under the HLGY condition, the HLG sources (left and right headlights of the oncoming car) were positioned at (4.6°H, -2.3°V) and (3.5°H, -2.3°V), respectively, relative to the visual acuity and contrast sensitivity letter position. The luminance of the light from the simulated left and right headlights (from the participant’s viewpoint) were 809 cd/m^2^ and 922 cd/m^2^, respectively. The luminance values of the simulated headlights (LED lights) were measured at the nominal participant viewpoint in the driving simulator (73 cm from the center of the simulator screen) using a Minolta LS-100 luminance meter (Konica Minolta, Inc., Osaka, Japan).

### Dynamic Scenario and Tasks

Two introductory and three dynamic nighttime driving scenarios were developed for the driving simulator (LE-1500 from FAAC, Inc., Ann Arbor, MI, United States). Each introductory scenario was about 12-min long, during which the participants were introduced to the general driving simulator features (e.g., target pedestrians, pedestrian motion, headlight simulation) and task (e.g., honk as soon as possible when a target pedestrian is detected), and provided a period during which participants adjusted to the operation of the driving simulator. During the introductory driving, participants also established a lack of visually induced motion sickness, which often occurs in driving simulators (Classen et al., 2011). The introductory scenarios were repeated if requested. No data was recorded during the introductory drives.

**FIGURE 1:**
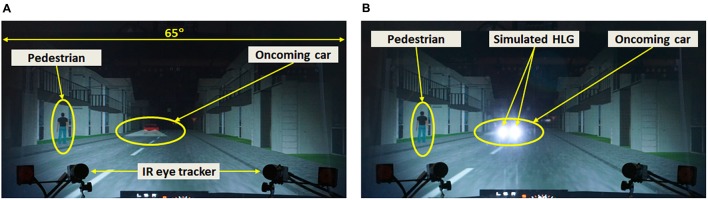
Photo of a pedestrian event (pedestrian walking-along left) with an oncoming car, with **(A)** the HLG simulator turned off, and **(B)** the HLG simulator turned on. When an oncoming car appears, a pedestrian appears on either the left or right side of the road, and then walks along or across the road.

All driving scenarios were driven in a dark room where all the room lights were turned off. The scenarios simulated urban nighttime driving conditions without streetlights, where the only light sources were the participant’s car headlights, limiting participant’s visibility to about ± 34° from car heading, restricting visibility to the front monitor (see **Figure 1**).

**FIGURE 2:**
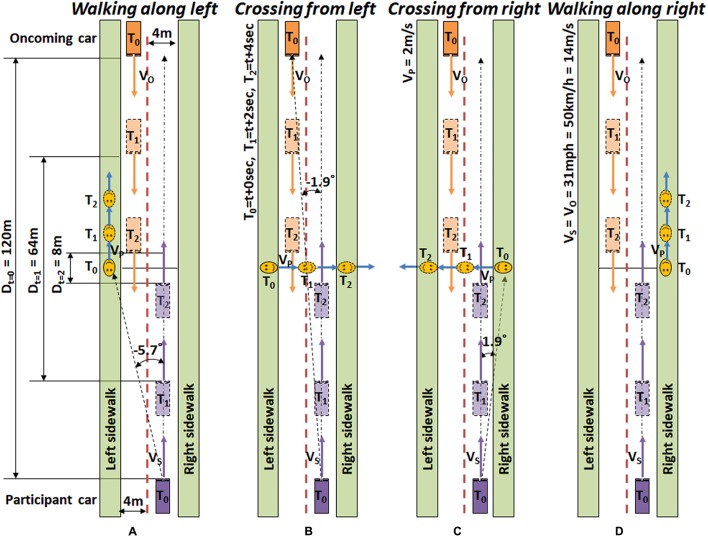
Timeline schematics of target pedestrian encounters. **(A)** A pedestrian appears on the left, and then walks along in the same direction as the participant car (*Walking along left*). **(B)** A pedestrian appears on the left, and then walks across the road from the left (*Crossing from left*). **(C)** A pedestrian appears on the right, and then walks across the road from the right (*Crossing from right*). **(D)** A pedestrian appears on the right, and then walks along the right sidewalk (*Walking along right*). Events are designed based on city driving speed (31 mph ≅ 50 km/h ≅ 14 m/s).

The three dynamic driving scenarios (about 12 min long each) were designed to simulate general nighttime driving at about 30 mph in urban or heavily settled areas. These scenarios contained intersections and undivided two or four lane roads, and each scenario contained 30 HLG events, where 24 of them included actual pedestrian encounters, and 6 of them were null-pedestrian encounters (no pedestrian appears). The pedestrians appeared on either the left or right side of the road, and then either walked along in the same direction as the participant’s car or crossed the road. Crossing and walking roadside are the two most common pedestrian behaviors that precede nighttime accidents (Sullivan and Flannagan, 2007). Those 4 types of pedestrian encounters (6 encounters of each type) were randomly distributed throughout each scenario. **Figure 2** shows the schema of pedestrian behaviors in such drives (assuming car speed of 31 mph) with oncoming traffic. Even if a pedestrian crosses the road from the left side (**Figure 2B**) the participant’s car will not crash with the pedestrian, as long as the participant keeps the speed under 44.7 mph. Pedestrians always cross the driver’s path at least 8 m in front of the participant’s car.

**FIGURE 3:**
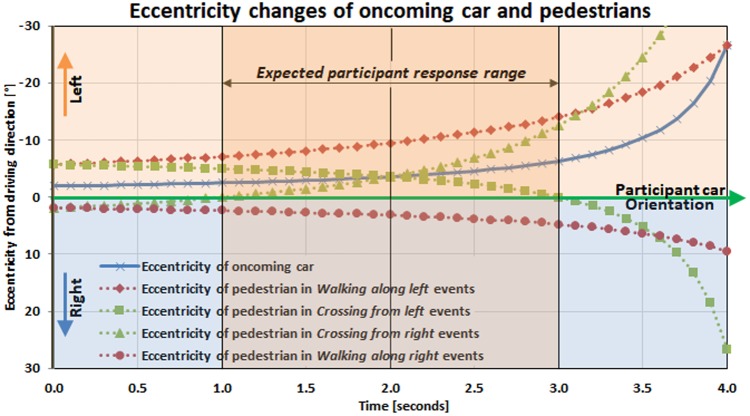
Temporal eccentricity changes of pedestrians and oncoming car relative to the participants’ driving direction. For the pedestrian crossing events, *Crossing from left* and *Crossing from right*, the pedestrian crosses the line between oncoming headlight and driver (participant) 2 s from the start of the event. For the pedestrian walk along events, *Walking along left* and *Walking along right*, pedestrian never crosses the oncoming car-to-participants line of sight during the 4 s event.

The initial pedestrian appearance eccentricity in left side events (*Walking along left* and *Crossing from left*) and right side events (*Walking along right* and *Crossing from right*) are -5.7°H and 1.9°H, respectively. The oncoming car initially appears at -1.9°H, which makes the proximities of initial pedestrian positions to the HLG source -3.8°H (for left) and 3.8°H (for right). As the participant’s car advances, the eccentricity of the pedestrian in the walking along events increases, while the eccentricity of pedestrians in the crossing events decreases and then increases, as shown in **Figure 3**.

In *Crossing from left* events, the pedestrians pass the oncoming car-to-subject line of sight 2 s after their initial appearance, and then cross the middle of the subject’s car lane at 3 s. The pedestrians in *Crossing from right* events cross the middle of the subject’s car lane at 1 s, and then start to cross the oncoming car-to-subject line of sight at 2 s. In *Walking along right* events, the pedestrians never approach the oncoming car-to-subject line of sight because they stay in the right visual field and move toward higher eccentricity. The eccentricity of the pedestrians in *Walking along left* events becomes closest to the oncoming headlight eccentricity at 4 s, but at this point, they move behind the oncoming car, so they never cross the oncoming car-to-subject line of sight. Note that regardless of which side the pedestrians start crossing, they cross the line of sight to the oncoming HLG at 2 s in the event timeline (**Figure 3**).

When a pedestrian appears 60 m away, the pedestrian spans about 0.5°H × 2°V and increases in size as the participant’s car approaches. During each oncoming HLG event, luminance and eccentricity of the oncoming HLG source increases from around 500 cd/m^2^ to 7,500 cd/m^2^, and its eccentricity varies from around 2° to 30° as the oncoming car passes by the participant’s car. The peak HLG luminance is reached when the oncoming car is about 8 m away from the subject car, where the oncoming car’s left and right headlights are located at (-26.0°H, -8°V) and (-13.4°H, -8°V), respectively (Hwang and Peli, 2013). At this peak HLG time, the crossing pedestrians reach the opposite sidewalk, while walking along pedestrians are either behind the oncoming car (*Walking along left*), or are positioned far to the right at 18°H (*Walking along right*).

The participant’s task was to drive the predefined path following the (scripted) directional voice cues, while adhering to normal traffic rules and regulations, and to honk the horn whenever he/she sees a pedestrian crossing or walking along the road.

### Procedures

On the first visit, participants’ visual acuity and visual field were measured in a standard clinical room to verify that the participants met the inclusion criteria (binocular visual acuity better than 20/40 for normal vision subjects, and 20/70 for real cataract patients, with habitual correction, and horizontal visual field wider than 120°). Monocular and binocular visual acuity were measured at 6 m viewing distance with negative polarity letters (black letters on white background) using the Test Chart 2000 Pro (Thomson Software Solutions, Hatfield, United Kingdom). A Goldmann visual field perimetry (V4e) verified the visual field requirement. Participants completed a pre-study questionnaire that evaluated the perceived level of HLG disability in real-world experiences. Participant’s visual acuity and letter contrast sensitivity were then measured in the driving simulator, seated at a comfortable driving position, with positive polarity letters (white letter on black background), while the stationary scenario was activated with the HLG simulator turned on and off. Finally, subjects drove the introductory scenarios and then completed three dynamic scenarios in both HLG simulation conditions (HLGN and HLGY), for each of the three clip-on conditions (plano lens, optically blurred, and simulated cataract), while the real cataract patients only drove in the two HLG conditions (HLGN and HLGY). The order of HLG conditions and dynamic scenario runs were counterbalanced among subjects.

On the second visit, participants drove an introductory scenario and then completed any remaining combinations of dynamic scenarios and vision conditions. Finally, the participants completed a post-study questionnaire regarding the perceived difficulty of the HLG in the driving simulator (as compared to real-world HLG). During the simulator driving sessions, participants were instructed to respond to the oncoming HLG as they would normally do in a real-world driving situation. The participants also completed a second HLG questionnaire composed of questions about the driver’s demographics, driving habits (Owsley et al., 1999), perceived HLG disturbance in the real world (Singh and Perel, 2004), and the difficulty of our simulated HLG encounters.

### Data Analysis

All simulator data was recorded for offline analyses, including scripted object data (e.g., oncoming car and pedestrian position and orientation), and subject’s car data (e.g., position, orientation, speed, brake, steering wheel rotation, and horn honks). However, only the detection time (time between pedestrian appearance and honk) was analyzed and reported here. Normal vision subjects drove the same three scenarios (in different orders) for all combinations of HLG and vision conditions, which allowed us to conduct a within-subject analysis. The detection time averages of each subject (based on six pedestrian encounters per condition) for all combinations of vision conditions (plano lens/optically blurred/simulated cataract), HLG conditions (HLGY/HLGN), and pedestrian types (*Walking along left*, *Walking along right*, *Crossing from left*, and *Crossing from right*) were computed. The resulting average response times were entered into a three-way ANOVA.

In addition to response time analysis, we calculated whether participants could have stopped in time to avoid a potential collision with the pedestrian. As described in **Figure 3**, each event in the driving scenarios was designed to last 4 s (e.g., it took 4 s for a pedestrian to cross the road). In *Crossing from left*, it took 2 s for pedestrians to enter the participant’s car lane. Since the usual braking distance for a car driving at 31 mph (14 m/s) is 45 ft (13.72 m), as we assume that the subject car’s mean braking deceleration is about 6 m/s^2^ (Fambro et al., 1997; AASHTO, 2011), a complete stop of the car would take about 2.3 s after hitting the break. Therefore, trials in which the pedestrian response took longer than 1.7 s from pedestrian appearance were considered ‘*untimely*’ (risky) responses, while the response times shorter than 1.7 s were considered ‘*timely*’ responses. In real world driving, drivers have additional options to maneuver the car to avoid a collision, so the safety threshold for the response time can be larger than 1.7 s but it should still be considered a risky event.

The pedestrians in *Walking along left* events walked along the oncoming car’s sidewalk (the other side of the road), so they were not considered as collision hazards for the participants. In the *Crossing from right* events, the pedestrians entered the subject’s car lane right after they appeared, and after 2 s, they left the participant car lane. Therefore, the pedestrians in the *Crossing from right* events were never a real collision hazard either. However, the pedestrians in *Walking along right* events walked along the subject’s car lane with close proximity, representing pedestrians walking in the participant’s lane during the event, so they had to be detected early and monitored throughout the event to avoid potential step-in toward the lane and collision. These pedestrians represent the real-world pedestrians walking along the road, where there is no sidewalk, or the sidewalk is covered with snow. Based on these assumptions, we analyzed the impact of the HLG on *timely* or *untimely* responses only for *Crossing from left* and *Walking along right* events, excluding the *Crossing from right* and *Walking along left* events. Note that although those data were excluded for the *timely*/*untimely* analysis, they were included in the response time and pedestrian miss analyses.

## Results

### Response Time for Pedestrian Detection

**FIGURE 4:**
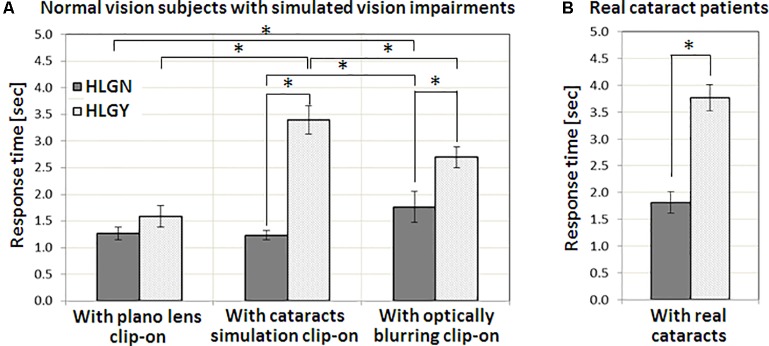
The impact of HLG on **(A)** normal vision subjects with three vision conditions, with a plano lens, simulated cataract, and optically blurring clip-ons, and on **(B)** patients with real cataracts. In both with simulated cataract and optically blurred conditions, oncoming HLG significantly increased response time for the pedestrians. The amount of response time delay with the presence of HLG for real cataract patients was similar to the simulated cataract condition. Significant differences in response time between conditions are marked by an asterisk (^∗^). Error bars represent the standard error within each group.

**FIGURE 5:**
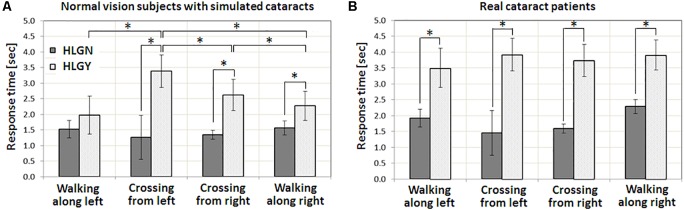
Impact of oncoming HLG on response time for various pedestrian types: pedestrian crossing from left and right, and walk along left and right. **(A)** Normal vision subjects with simulated cataracts. **(B)** Patients with real cataracts. The main effect of pedestrian types and the interaction between HLG conditions and pedestrian types were found to be significant for normal vision subjects with simulated vision impairments. However, neither was significant for patients with real cataracts. Significant increases in response time are marked by an asterisk (^∗^). Error bars represent the standard error within each group.

**Table 2** summarizes the averages and standard deviations of response times for all vision conditions, pedestrian types, and HLG conditions. Statistically significant differences between HLGN and HLGY are shown graphically in **Figure 4**. The significant differences between simulated and real cataracts vision conditions are shown in **Figure 5**. A significant main effect of HLG [*F*(1,96) = 88.86, *p* < 0.01] was found for normal vision subjects with simulated impaired vision conditions (**Figure 4A**), where the average response time under HLGY (2.56 ± 1.25 s) was substantially longer than under HLGN (1.42 ± 0.50 s). A significant main effect of vision conditions was also found [*F*(2,96) = 21.80, *p* < 0.01], where the response time with simulated cataract clip-ons (2.32 ± 1.34 s) and with optically blurred clip-ons (2.23 ± 1.03 s) were significantly longer than with plano lens clip-ons (1.43 ± 0.62 s) [all *t*s(39) > 4.76, *p*s < 0.01]. The average performance (combining HLGY and HLGN) with optically blurred clip-ons was not significantly different from the performance with simulated cataract clip-ons [*t*(39) = 2.02, *p* = 0.68] (**Figure 4A**). However, the response times with optically blurred clip-ons were significantly longer under HLGN [*t*(19) = 3.40, *p* < 0.01], but significantly shorter under HLGY [*t*(19) = 2.16, *p* = 0.04] than the response times with simulated cataract clip-ons.

**Table 2:** Response times for all vision conditions, pedestrian types, and HLG conditions.

	Normal vision subjects with simulated vision impairments						Patients with real cataracts	
	With plano lens		Simulated cataracts		Optically blurred			
	HLGN	HLGY	HLGN	HLGY	HLGN	HLGY	HLGN	HLGY
Walk-along left	1.39 ± 0.43	1.22 ± 0.17	1.22 ± 0.08	2.09 ± 0.52	1.94 ± 0.78	2.62 ± 1.90	1.92 ± 0.63	3.50 ± 1.38
Cross-from left	1.17 ± 0.17	2.57 ± 0.12	1.05 ± 0.18	4.42 ± 0.52	1.57 ± 0.36	3.15 ± 0.78	1.46 ± 0.31	3.92 ± 1.12
Cross-from right	1.23 ± 0.20	1.27 ± 0.23	1.21 ± 0.16	4.18 ± 0.26	1.60 ± 0.45	2.41 ± 0.54	1.59 ± 0.50	3.74 ± 1.04
Walk-along right	1.28 ± 0.15	1.30 ± 0.34	1.46 ± 0.39	2.90 ± 0.55	1.94 ± 1.03	2.62 ± 1.06	2.28 ± 1.57	3.91 ± 1.16
Average of all events types	1.27 ± 0.26	1.59 ± 0.81	1.23 ± 0.26	3.40 ± 1.07	1.76 ± 0.67	2.70 ± 1.13	1.81 ± 0.89	3.77 ± 1.10

*Shown as average ± standard deviation of response times in seconds*.

Significant two-way interactions were also found between HLG and vision conditions [*F*(2,96) = 19.96, *p* < 0.01], where the performance reduction caused by HLGY was largest with simulated cataract clip-ons [all *t*s(19) > 3.66, *p*s < 0.04], followed by optically blurred clip-ons [*t*(19) = 2.23, *p* = 0.04]. The oncoming HLG did not cause any significant performance reduction with plano lens clip-ons [*t*(19) = 1.75, *p* = 0.10]. These results (**Figure 4A**) suggested that the performance with our simulated cataract clip-ons was affected more by the presence of oncoming HLG (as the increase of response time with plano lens clip-ons and simulated cataract clip-ons were 0.32 and 2.17 s, respectively) due to the scattering of light. Note that the performance with plano lens clip-ons and with simulated cataract clip-ons under HLGN were not significantly different [*t*(19) = 0.54, *p* = 0.59], but the performance with optically blurred clip-ons under HLGN was significantly worse than with plano lens clip-ons and with simulated cataract clip-ons [all *t*s(19) > 3.40, *p* < 0.01]. However, the effect of HLG condition (increase of the response time) was much larger with simulated cataract than with optically blurred clip-ons (as the response time increases with simulated cataract and optically blurred were 2.17 and 0.94 s, respectively). No significant three-way interaction among HLG conditions, vision conditions, and pedestrian types was found [*F*(6,96) = 1.51, *p* = 0.18].

For normal vision subjects with simulated cataract clip-ons, a significant main effect of HLG [*F*(1,39) = 332.27, *p* < 0.01] was found, where the average response time under HLGY was substantially longer than the response time under HLGN. A significant main effect of pedestrian type was also found [*F*(3,39) = 18.28, *p* < 0.01], where the response times for the pedestrians in *Crossing from left* and *Crossing from right* events were significantly larger than for the pedestrians in *Walking along left* and *Walking along right* events [all *t*s(29) > 2.11, *p* < 0.04] (**Figure 5A**). Response times under HLGN for each pedestrian type were not significantly different from each other [all *t*s(29) < 1.10, *p*s > 0.28]. Under HLGY, the response times for the pedestrians in *Crossing from left* and *Crossing from right* events were not significantly different [*t*(4) = 0.79, *p* = 0.47], but they were significantly longer than for the pedestrians in *Walking along left* [all *t*s(4) > 3.57, *p*s < 0.02] and *Walking along right* events [all *t*s(4) > 6.77, *p* < 0.01]. Interactions between HLG and pedestrian types were found to be significant [*F*(3,32) = 25.63, *p* < 0.01], where the significant performance reduction due to the HLG was more apparent for the pedestrians in *Crossing from left* [3.37 s, *t*(14) = 7.28, *p* < 0.01] and *Crossing from right* [2.97 s, *t*(14) = 3.70, *p* = 0.00] events, followed by the pedestrians in *Walking along right* [1.44 s, *t*(14) = 3.49, *p* < 0.01], and *Walking along left* [0.87 s, *t*(14) = 1.25, *p* = 0.23] events.

For real cataract patients, a significant main effect of HLG [*F*(1,39) = 40.74, *p* < 0.01] was also found, where the average response time under HLGY (3.77 ± 1.10 s) was substantially increased from the response time under HLGN (1.81 ± 0.89 s) (**Figure 4B**). However, unlike the simulated cataract condition, no significant main effect of pedestrian type was found [*F*(3,39) = 0.43, *p* = 0.73]. Also, no significant interaction between HLG conditions and pedestrian types [*F*(3,32) = 0.82, *p* = 0.43] was found. The negative effects of oncoming HLG (delay of response time by HLG) were significant [all *t*s(5) = 4.57, *p* < 0.01] for all pedestrian types, where the response times for the pedestrians in *Crossing from left*, *Crossing from right*, *Walking along right*, and *Walking along left* were 2.46, 2.15, 1.63, and 1.58 s, respectively (**Figure 5B**).

### Failures of Pedestrian Detection and Untimely Responses

Under HLGY, the normal vision subjects with the plano lens clip-on missed (failed to respond to the pedestrian) only one event throughout all drives (0.5%, in *Walking along left*). The same group with the simulated cataract clip-on missed 12 events (6%: 2 *Walking along left*, 2 *Crossing from left*, 6 *Crossing from right*, and 2 *Walking along right*). Under HLGN, no event was missed with plano lens clip-on, and only one event (0.5%, in *Walking along right*) was missed with simulated cataract clip-on. For real cataract patients, 15% of events (16 *Walking along left*, 12 *Crossing from left*, 9 *Crossing from right*, and 17 *Walking along right*) were missed under HLGY, and 6% of the pedestrian events (3 *Walking along left*, 0 *Crossing from left*, 1 *Crossing from right*, and 8 *Walking along right*) were missed under HLGN. However, one of the patients with cataracts (RC1) performed worse than others, and the majority of the misses were accounted for that patient (62% and 90% of all misses under HLGY and under HLGN, respectively). With that particular patient excluded from the analysis, the percent missed dropped to 8% under HLGY and 1% under HLGN, similar to the percent missed with simulated cataract clip-on condition. Note that this particular patient is the oldest (75 years old).

When the 1.7 s untimely response threshold (described in *Data analysis*) was applied for the response times for the pedestrians in *Crossing from left* and *Walking along right* events, we found that 34 (19%) untimely responses occurred under HLGY, and just 9 (5%) untimely responses under HLGN. With simulated cataract clip-on, 79 (44%) untimely responses occurred under HLGY, but just 19 (11%) untimely responses occurred under HLGN. These indicate that the presence of oncoming HLG increased the number of untimely responses for both subjects with plano lens and simulated cataract clip-ons, but the increase with simulated cataract clip-on was larger than with plano lens clip-on (14% increase with plano lens and 33% increase with simulated cataract). For real cataract patients, 164 (91%) untimely responses occurred under HLGY, but 72 (40%) untimely responses under HLGN were found. Again, the same real cataract patient (RC1) failed to respond to almost all pedestrian events even for HLGN (as well as under HLGY). Excluding that particular patient’s data from the analysis resulted in 128 (71%) and 42 (23%) untimely responses under HLGY and HLGN, respectively (48% increase with HLG). Note that the missed pedestrian events were also counted as untimely responses.

### HLG Questionnaire

**FIGURE 6:**
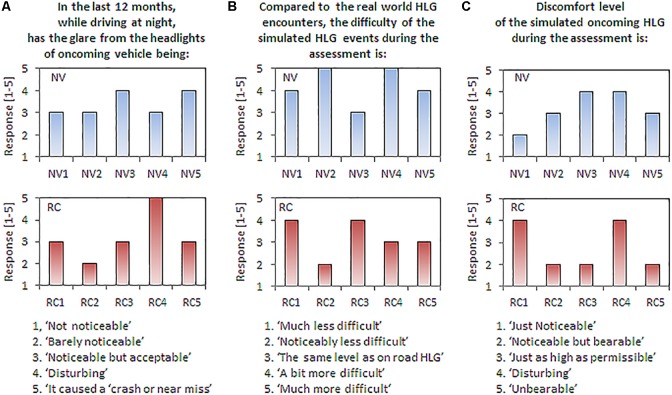
Responses to the questionnaires by normal vision subjects (top row) and patients with real cataracts (bottom row). The questions are shown on the top and scaled responses are shown on the bottom. **(A)** Perception of real-world HLG during nighttime driving. **(B)** Comparison of difficulty between the simulated and real-world HLG encounter events. **(C)** Discomfort level of the simulated HLG during the assessments.

Two out of the five normal vision subjects were current drivers, and the three non-current drivers had stopped driving within the last 2 years (living in a city). All had driving experience of 2.7 ± 1.9 years. They drove 3.0 ± 2.9 days per week where about 70% was nighttime driving (2.1 ± 1.9 days per week). Four out of five normal vision subjects stated that the nighttime driving is more difficult than daytime driving. All normal vision subjects rated the disturbance of real-world oncoming HLG as ‘*noticeable but acceptable*’ or ‘*disturbing*’ (3.27 ± 0.55 on 1-to-5 scales, **Figure 6A**, top). Most subjects ranked our simulated oncoming HLG events as more difficult than the perceived difficulty of real-world HLG encounters (4.2 ± 0.8 on 1-to-5 scales, **Figure 6B**, top). The discomfort level of the simulated oncoming HLG during the assessments was rated between ‘*just as high as should be permissible*” and ‘*disturbing*’ (3.2 ± 0.8 on 1-to-5 scales, **Figure 6C**, top). Note that these responses were gathered after the subjects finished all drives with all vision conditions, including the simulated cataracts condition.

All five patients with real cataracts were current drivers with 50 ± 4.7 years of driving experience and they drove about 5.9 ± 1.1 days per week, where about 46% of weekly drives include nighttime driving (2.7 ± 1.6 days per week). The difficulty of nighttime driving in the real world for the patients with real cataracts was similar to the normal vision subjects’ rating, where four out of five patients stated that the nighttime driving is more difficult than daytime driving. The disturbance of HLG in real-world oncoming HLG was also similarly rated as the normal vision subject’s rating, but one patient reported ‘*it caused a crash or near misses*’ (3.2 ± 0.84, **Figure 6A**, bottom). The patients rated our simulated HLG encounters as about the same level of difficulty as the similar events experienced in real world (3.2 ± 0.8, **Figure 6B**, bottom). Finally, the discomfort due to the simulated HLG during the assessments was rated slightly lower than real-world HLG (2.8 ± 1.1, **Figure 6C**, bottom). Patients with real cataracts rated the difficulty and discomfort of the oncoming HLG lower than normal vision subjects, but the differences were not significantly different [all *t*s(8) < 1.89, *p*s > 0.10].

### HLG Effect on Visual Acuity

**FIGURE 7:**
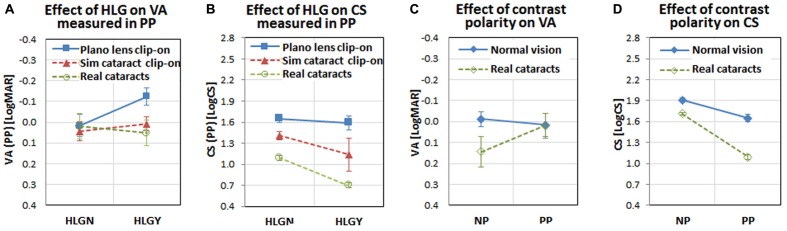
Interactions among visual functions, vision conditions, HLG conditions, participant groups, and measurement methods. Note visual acuity and contrast sensitivity were measured with both positive polarity letters (*PP*: bright letter on dark background) and negative polarity letters (*NP*: dark letter on bright background). **(A)** The interaction between visual acuity and HLG condition for vision conditions shows visual acuity improvement under HLGY with the plano lens (presumably due to the positive impact of pupillary myosis), but the effect of myosis disappeared for simulated cataract and real cataracts (presumably due to the negative impact of light scatter by the simulated and real cataracts). **(B)** The interaction between contrast sensitivity and HLG condition shows a larger negative impact of HLG on contrast sensitivity with simulated cataract and real cataract, compared to the slight negative impact with plano lens. **(C)** The interaction between visual acuity and measurement method shows that the different polarity of the target letter does not affect the normal vision subjects’ visual acuities, but does affect the real cataract patients (visual acuity decrease with negative polarity). **(D)** The interaction between contrast sensitivity and measurement method shows that both normal vision subjects and real cataract patients’ contrast sensitivities are affected similarly by the letter polarity (both reduced with positive polarity). The error bars represent the standard errors within each group.

The averages and standard deviations of binocular visual acuity of the normal vision subjects with plano lens clip-on under HLGN and HLGY were 0.02 ± 0.12 and -0.12 ± 0.09 LogMAR, respectively, and with simulated cataract clip-on under HLGN and HLGY were 0.04 ± 0.10 and 0.01 ± 0.08 LogMAR, respectively (**Figure 7A**). A (2 × 2) ANOVA for HLG conditions (HLGY/HLGN) × simulated vision conditions (plano lens/simulated cataract) was applied to the visual acuities for normal vision subjects. The effect of HLG approached significance [*F*(1,19) = 3.90, *p* = 0.07], where the visual acuity under HLGY was better than under HLGN. The effect of simulated cataract also approached significance [*F*(1,19) = 3.22, *p* = 0.09], where visual acuities with simulated cataract and with plano lens under HLGN were not significantly different [*t*(1,4) = 0.43, *p* = 0.69], but the difference between with simulated cataract and with plano lens was significant under HLGY [*t*(1,4) = 3.06, *p* = 0.04]. No significant interactions were found between HLG and vision conditions [*F*(1,16) = 1.36, *p* = 0.26].

For the real cataract patients, the visual acuities measured under HLGN and HLGY were not significantly different [*t*(1,4) = 1.97, *p* = 0.12]. The average and standard deviation of visual acuities under HLGN and HLGY were 0.06 ± 0.05 and 0.04 ± 0.07 LogMAR, respectively (**Figure 7A**). A 2 × 2 between-subjects ANOVA was applied for HLG condition (HLGY/HLGN) × cataract group (real cataract/simulated cataract). No significant effect was found for HLG condition [*F*(19,1) = 0.00, *p* = 0.97], vision condition [*F*(19,1) = 0.04, *p* = 0.84], or the interaction between vision and HLG conditions [*F*(16, 1) = 0.43, *p* = 0.52], indicating that the impact of HLG on visual acuity of the simulated cataract subjects was not different from the real cataract patients.

### HLG Effect on Contrast Sensitivity

The mean and standard deviation of binocular contrast sensitivities for the normal vision subjects with plano lens clip-on under HLGN and HLGY were 1.65 ± 0.12 and 1.59 ± 0.23 LogCS, respectively, and with simulated cataract clip-on for HLGN and HLGY were 1.41 ± 0.13 and 1.14 ± 0.53 LogCS, respectively (**Figure 7B**). The main effect of vision condition was significant [*F*(1,19) = 6.53, *p* = 0.02], where the contrast sensitivity with simulated cataract clip-on was lower than the contrast sensitivity with plano lens clip-on under HLGN [*t*(1,4) = 4.15, *p* = 0.01], as well as under HLGY [*t*(1,4) = 2.82, *p* = 0.05]. The effect of HLG condition was not significant [*F*(1,19) = 1.42, *p* = 0.25]. No significant interactions were found between HLG and vision condition [*F*(1,16) = 0.41, *p* = 0.53].

For the real cataract patients, a significant difference in contrast sensitivity was found between HLGN and HLGY conditions [*t*(1,4) = 11.76, *p* < 0.01]. The mean and standard deviation of contrast sensitivities for real cataract-HLGN and real cataract-HLGY conditions were 1.09 ± 0.08 and 0.70 ± 0.08 LogCS, respectively (**Figure 7B**). A significant main effect of vision condition [*F*(19,1) = 14.76, *p* < 0.01] was found, where the contrast sensitivity for real cataract was worse than simulated cataract. No significant main effect of HLG was found [*F*(19,1) = 2.87, *p* = 0.11]. No significant interaction was found [*F*(16,1) = 0.20, *p* = 0.66], indicating that the impact of HLG (reduction of contrast sensitivity due to HLG) was not different between the simulated cataract subjects and real cataract patients. Note that even though the mean binocular contrast sensitivity of the real cataract patients under HLGY and HLGN was poorer than that of simulated cataract subjects, the binocular visual acuity of the real cataract patients was similar to that of the simulated cataract subjects.

### Effect of Different Methods of Measuring Visual Functions

The participants’ visual acuity and contrast sensitivity were measured in two conditions, once in a conventional clinical condition under room illumination with negative polarity letters (dark letter on bright background), and the other in the driving simulator under dark room condition with positive polarity letters (bright letter on dark background). To determine the impact of the letter polarity on visual function measures for different participant groups (normal vision subjects and real cataract patients), a pairwise *t*-test was applied between the negative polarity measurements (visual acuities and contrast sensitivities in **Table 1**) and positive polarity measurements (visual acuities and contrast sensitivities measured with plano lens clip-on under HLGN).

For real cataract patients, the increase of visual acuity measured in positive polarity approached significance [*t*(4) = 2.28, *p* = 0.07], but for normal vision subjects visual acuity was not significantly different when measured in positive polarity [*t*(4) = 0.49, *p* = 0.65]. The visual acuities measured in negative and positive polarity for normal vision subjects were -0.012 ± 0.006 and 0.016 ± 0.014, respectively, and for real cataracts patients, they were 0.144 ± 0.027 and 0.020 ± 0.018 LogMAR, respectively (**Figure 7C**). For contrast sensitivity measures, a significant decrease was found with positive polarity letters, compared to the measurements with the negative polarity for both normal vision subjects [*t*(4) = 4.06, *p* = 0.02] and real cataract patients [*t*(4) = 12.04, *p* < 0.00]. The contrast sensitivities in negative and positive polarity for normal vision subjects were 1.91 ± 0.02 and 1.65 ± 0.01 LogCS, and for the real cataract patients, they were 1.71 ± 0.01 and 1.09 ± 0.01 LogCS, respectively (**Figure 7D**). Similar results were also reported in our previous studies (Hwang and Peli, 2015, 2016), where the visual acuity was not significantly affected by polarity of the presented letter, but the contrast sensitivity was significantly affected (contrast sensitivity is reduced when measured in positive polarity).

### Correlation Between Vision Measures and Response Time

A Pearson correlation coefficient for each subject group (normal vision/real cataract) was computed between: (1) visual acuity and contrast sensitivity measured in clinical condition and response time measured under HLGN, (2) visual acuity and contrast sensitivity measured in negative polarity and response time measured under HLGY, (3) visual acuity and contrast sensitivity measured in dark room condition under HLGN and response time measured under HLGN, (4) visual acuity and contrast sensitivity measured in positive polarity under HLGY and response time measured under HLGY, (5) change in visual acuity and contrast sensitivity in positive polarity due to HLG and response time change due to HLG. The correlation between the contrast sensitivity of simulated cataract measured in positive polarity under HLGY and response time of simulated cataract under HLGY was found to be significant (*p* = 0.03). However, scatter plot indicates that these significant correlations were strongly governed by an extreme data point. No other significant correlation (all *p*s > 0.2) was found, regardless of vision measures (e.g., visual acuity and contrast sensitivity) and measurement conditions (negative and positive polarity).

### Parametric vs. Non-parametric Analysis

We also applied Shapiro–Wilk tests to the response times for normal vision subjects and real cataract patients to test the data for normality. The results indicated that the majority of data for each of the vision-HLG conditions are not normally distributed, while the data for plano lens under HLGN, and simulated cataracts under both HLGY and HLGN were normally distributed. Therefore, Wilcoxon signed-rank test (instead of the paired *t*-test) and Kruskal–Wallis test (in place of the parametric ANOVA) were applied to test the HLG effect for all viewing-HLG conditions. However, those non-parametric analysis resulted in the same conclusions as described in the results section (except the one that normal vision subjects with plano lens made a significant delay with the presence of oncoming HLG, which was rejected by non-parametric analysis). Therefore, the parametric analysis results were shown here for its robustness.

## Discussion

Our study measured subjects’ driving behaviors and included simulation of dynamic oncoming HLG with walking pedestrians. The driving scenarios focused on detection of pedestrians (non-illuminating and non-retroreflective hazards), which are the main collision risks during nighttime driving. We have shown that the impact of oncoming HLG is measurable with this protocol, even with mild cataracts, and may be highly consequential. We also found that response times to pedestrians walking along the road on either side or crossing the road from either side is significantly impacted by the presence of oncoming HLG. The presence of HLG increased the patients’ response times to such an extent that significantly more pedestrians were detected too late to avoid a collision, and a few were never detected.

Using simulated vision impairments, we were able to study the impact of HLG within subjects, controlling for confounds such as driving experience and individual response time difference. Comparing the performances between simulated cataract and optically blurred conditions enabled us to examine the impact of light scatter and impact of blur separately, where both reduced visual acuity. We found that the performance with simulated cataract was more affected by the presence of oncoming HLG than with optically blurred lenses, even though both caused a similar loss of acuity under the HLGN condition. This suggests that the impact of HLG on performance with simulated cataract was due to the light scatter characteristics of the cataract simulation. This conclusion is strengthened by the finding that the response time with optically blurred lenses was longer than with simulated cataract under HLGN condition, but shorter under HLGY condition.

We expected the response time with optically blurred clip-ons under HLGN to be similar to the performance with simulated cataracts because the plus lens clip-ons worn by the subjects in the optically blurred condition reduced visual acuity by a similar amount as the simulated cataract clip-ons. However, our data showed that the response times with optically blurred clip-ons were significantly longer than in other vision conditions under HLGN. This finding indicates that the detection performance was not directly related to visual acuity.

Because the eccentricities of the pedestrians on the right side events were lower (especially for the first 2 s) (**Figure 3**), we expected the pedestrians appearing on the right side (*Crossing from right* and *Walking along right*) to be detected earlier than the corresponding events on the left side (*Crossing from left* and *Walking along left*) under HLGN. However, the response times were not different among pedestrian types under HLGN (**Figure 5A**), indicating that the eccentricity differences between the left and right pedestrians (**Figure 3**) were insufficient to affect performance because both pedestrian types were still close enough to the subject’s car path (within ± 10°H) that they could be detected.

Under HLGY, the response times for crossing pedestrians (*Crossing from left* and *Crossing from right*) were longer than for walk along pedestrians (*Walking along left* and *Walking along right*) (**Figure 5A**). This was the case despite the fact that the animation of the pedestrian (biological motion) was more visually distinct from the side (in crossing events) than from the back (in walking along events), and the walk-along pedestrians’ eccentricities to the participant’s car direction were larger than that of the crossing pedestrians. Relative to the glare source, the average angular proximities of the pedestrian for *Crossing from left* and *Crossing from right* for the first 2 s of an event were 2.27 ± 1.16° and 2.28 ± 1.16°, respectively, while the average proximity for *Walking along left* and *Walking along right* pedestrians were larger, 4.71 ± 0.64° and 5.02 ± 0.90°, respectively (**Figure 3**). The longer response times for crossing events than walking along events suggests that the pedestrian’s proximity to the glare source plays a dominant role in response time.

The pedestrian type was a significant factor in the performance change under HLGY for the simulated cataract subjects, but not for the real cataract patients. This result may suggest that the pedestrian proximity to the glare source was not a factor for the real cataract patients. However, if one considers the difference in vision measures (especially contrast sensitivity) between normal vision subjects and the real cataract patients (**Figure 7B**), an alternate interpretation might be that the veiling glare caused by the oncoming HLG further reduces the patients’ contrast sensitivity (**Figure 7D**) to such an extent that the smaller effects of the pedestrian types and their distance from the glare source was overwhelmed by the stronger effect of HLG.

The lack of correlation between visual function measures and response time suggests a general limitation of conventional vision measures for predictions of the impact of oncoming HLG on driving performance, as identified in prior studies (Chylack et al., 1993; Featherstone et al., 1999). Another study (Ginsburg, 1987) suggested that contrast sensitivity might be a more relevant measure than visual acuity for predicting night driving safety, but our data did not support this claim.

Compared to the previous simulated vision study (Wood et al., 2012), our study included a large number of (non-null) pedestrian encounters (a total of 72 scored encounters for each participants for each vision condition) under each HLG condition, where only 2 pedestrian encounters were included per drive for two drives (a total of 4 per subject) in their study. As a result, our design was more robust to subject mistakes. Even with the increased number of pedestrian encounters, the participants (especially the real cataract patients) reported that our HLG experiments closely simulated the difficulty of real-world HLG events (**Figure 6B**). Importantly, the reduction of the pedestrian detection and response time in our data due to the presence of HLG with simulated cataract was comparable with the performance that we measured with the real cataract patients, which indicates our simulated cataract clip-on reasonably simulated the performance reduction of real cataract under HLG.

Our data indicated that the oncoming HLG negatively affected all vision condition groups, but, as expected, the effect was bigger for the simulated cataract and real cataract conditions. Our results also suggest that conventional clinical vision measures such as visual acuity and contrast sensitivity have limited predictive power for estimating the impact of oncoming HLG on nighttime driving performance. Even with just 5 participants in each group, this pilot study demonstrated that the impact of oncoming HLG with cataracts on hazard detection performance could be effectively measured.

Currently, we are applying the same experimental paradigm to measure changes in the impact of oncoming HLG on patients with bilateral cataracts as the patients undergo cataract surgeries for both eyes. An important issue to be addressed in that study is whether untreated cataracts in one eye may negatively affect overall driving performance with HLG. This has been identified as a possibility by Owsley et al. (2001) based on retrospective analysis of crash records of cataract patients. This analysis, however, did not separately analyze day and nighttime accidents.

## Author Contributions

AH put his contribution towards the study design, data collection, data analysis, and writing of the manuscript. MT-B worked on data collection and data analysis, and RG worked on data analysis and study design. EP contributed to the study design, data analysis, and writing of the manuscript, and he also supervised general study practice. All four had regular meetings to discuss in this study.

## Conflict of Interest Statement

The authors declare that the research was conducted in the absence of any commercial or financial relationships that could be construed as a potential conflict of interest.
